# **Development of systemic sclerosis in patients with autoimmune hepatitis: an emerging overlap syndrome**

**Published:** 2016

**Authors:** Roberto Assandri, Marta Monari, Alessandro Montanelli

**Affiliations:** 1 *Clinical investigation laboratory, Humanitas Clinical and Research Center, Via Alessandro Manzoni 56 20089 Rozzano (Miano) Italy*; 2 *Clinical Laboratory, Diagnostics Department, Spedali Civili of Brescia, Piazzale Spedali Civili 1 - Brescia, Italy *

**Keywords:** Autoimmune liver diseases, Systemic sclerosis, Autoimmune hepatitis

## Abstract

**Aim:**

We described two case reports of AIH/SSc overlap syndrome and reviewed literatures regarding this issue.

**Background:**

AIH is a chronic hepatitis of unknown aetiology characterized by continuing hepatocellular necrosis and inflammation. AIH overlap syndromes have been reported with other autoimmune diseases.

**Patients and methods:**

According to the classification criteria for SSc, we conducted a retrospective chart review of 35 cases with biopsy-proven AIH over the past 5 years at our institution. We reviewed the MEDLINE database using the appropriate key-words.

**Results:**

A chart review of 35 cases (M/F ratio 1:2, mean age 47.6±10.3 years) revealed nine patients (9/35, 25.7%) with CTD (four males and three females with a mean age of 45.1±8.4 years). All patients had ANA. Four patients were SSA/Ro positive UCTD (1/35, 2.85%), and six patients developed SLE (6/35, 17.1%). Only two female patients (2/35, 5.7%) with specific SSc AAb developed a systemic sclerosis. We described a patient with AIH who was diagnosed with diffuse systemic sclerosis-sine scleroderma with positive anti-centromere B and SSA/Ro52 KDa antibodies. We also reported a patient with AIH who was diagnosed limited SSc with contemporary presence of anti-centromere A and anti-RNA polymerase III antibody.

**Conclusion:**

We suggest that SSc may be considered to be one of the manifestations associated with AIH. Patients with AIH may have an increased risk to develop SSc and should be followed, especially when Raynaud phenomenon was found.

## Introduction

 The liver is a largest lymphoid organ involved in the immune response against pathogens and in the maintenance of tolerance to self-molecules (1). The liver can also be a target of an autoimmune reaction, as observed in primary liver autoimmune diseases, such as autoimmune hepatitis (AIH), primary biliary cirrhosis (PBC), and primary sclerosing cholangitis (PSC) (1). AIH is a chronic hepatitis of unknown etiology characterized by hepatocellular necrosis, inflammation, as well as the presence of autoantibodies and high serum gamma-globulin concentrations. Diseases that are commonly seen in patients with AIH, include autoimmune thrombocytopenia, type 1 diabetes, thyroiditis, and ulcerative colitis (1,2). However, systemic connective tissue diseases (CTD) such as systemic lupus erythematosus (SLE), undifferentiated connective tissue disease (UTCD), mixed connective tissue disease (MCTD) and Systemic sclerosis (SSc) have been infrequently associated with AIH (3,4). Therefore, only eleven cases of AIH with SSc have been reported in the literature. All these cases with SSc have limited clinical form and with only one exception, AIH occurs after the diagnosis of SSc (5-15).

Based on these evidences, we conducted a clinical and immunological chart review of 35 cases with biopsy-proven AIH over the past five years at our institution. We reported two cases with AIH/SSc overlap syndrome and briefly reviewed the literature. 

## Patients and Methods

**Patients**

We conducted a retrospective chart review of 35 cases with biopsy-proven AIH over the past five years at our institution. All patients had liver biopsies consistent with AIH. Other causes of hepatitis, including viral infections were excluded. The clinical charts were reviewed in the development of SSc, according to the classification criteria for SSc (13). 

**Literature review**

We reviewed the MEDLINE (National Library of Medicine, Bethesda, MD) database from 1950 to 2014, using and combining the following key-words: “Autoimmune hepatitis”, “connective tissue disease”, “SLE” and “systemic sclerosis”. Available articles were analyzed and only articles with a focus on the prevalence and clinical significance of AIH and SSc, as well as overlap syndrome were retained. 

## Results

A chart review of 35 cases (M/F ratio 1:2, mean age 47.6±10.3 years) with biopsy-proven AIH over the past 5 years at our institution revealed nine patients (25.7%) with CTD (four male and three females with a mean age of 45.1±8.4 years) (Table-1). All patients had ANA and four patients were SSA/Ro positive. One patient developed an undifferentiated connective tissue disease (UCTD) (1/35, 2.85%), and six patients developed SLE (6/35, 17.1%). Only two female patients (2/35, 5.7%) with specific SSc autoantibodies (AAb) developed a systemic sclerosis. 

**Patient-I**

A 70-year-old woman presented to our institution for a re-evaluation of disease status.

AIH had been diagnosed seven years earlier and she was treated with prednisone and azathioprine. At the time of diagnosis, abdominal ultrasonography revealed liver cirrhosis with splenomegaly and a moderate amount of ascites. Liver biopsy showed partial expansion with lymphoplasmacytic infiltration, bridging fibrosis, and multifocal drop out of hepatocytes, replaced by lymphoid cells in the lobules, suggesting chronic active hepatitis. According to the Knodell index, the histological grading was 11+4, and International Autoimmune Hepatitis Group score was 18. Viral serologies (hepatitis B surface antigen, anti-hepatitis B surface antigen, immunoglobulin G anti-hepatitis B core antigen, anti-hepatitis C virus, cytomegalovirus, Epstein-Barr virus, herpes simplex virus, and human immunodeficiency virus) were all negative. 

**Table 1:** Characteristics of patients with AIH

Patient No.	Age/Sex	CTD	ANA	ENA	Specific SSc AAb	dsDNA	Anti smooth muscle
1	51/M	SLE	1:640 Homogeneus	Neg	Neg	Neg	Pos
2	50/M	SLE	1:320 Speckled	Neg	Neg	neg	Pos
3	48/F	UCTD	1:320 Speckled	SSA/Ro52 KDa	Neg	Neg	Pos
4	52/M	SLE	1:1280 Homogeneus	SSA/Ro52 KDa	Neg	Pos	Pos
5	46/F	SLE	1:1280 Homogeneus	SSA/Ro60, Ro52 KDa	Neg	Pos	Pos
6	28/F	SLE	1:1280 Homogeneus	Neg	Neg	Pos	Pos
7	41/M	SLE	1:1280 Homogeneus	Neg	Neg	Neg	Pos

Abbreviations: F, female; M, male; SLE, systemic lupus erythematosus; UCTD, undifferentiated connective tissue disease, ENA, extraible nuclear antigens.

**Table 2:** Immunological features of patients with AIH and SSc

Author (Literature Ref)	Liver disease	CDT	ANA	ACA	SMA	LKM	AMA	dsDNA AAb
Ishikawa et al (6)	AIH	lSSc	+	+	+	ND	-	ND
Lis-Swiety et al (8)	AIH	SSc-PM	+	ND	+	-	-	ND
Marie et al (9)	AIH	lSSc	+	+	-	-	-	ND
Pamfil et al (11)	AIH	SSc-PM	+	+	-	-	-	-
Rodrigues et al (12)	AIH	dSSc	ND	ND	-	-	+	ND
Ngo Mandag et al (33)	AIH	lSSc	+	+	-	-	-	ND
Yabe et al (34)	AIH	lSSc	-	-	NK	NK	-	ND

CDT: connective tissue disease; ANA: antinuclear antibodies; ACA: anticentromere antibodies; SMA: anti-smooth muscle antibodies; LKM: anti-liver-kidney microsome-1 antibodies; AMA: antimitochondrial antibodies; lSSc: limited SSc; dSSc: diffuse SSc; SSc-PM: systemic sclerosis-polymyositis overlap syndrome; +: positive; -: negative; ND: not determined.

Based on the 6 months prior to the presentation, the patient had frequent episodes of Raynaud’s phenomenon. On physical examination, the patient did not present sclerodactyly, skin thickening, digital pitting scars, or digital edema and telangiectasia characterizing limited form of SSc. However, periungueal capillaroscopy showed nail fold capillary abnormalities and giant capillaries. High resolution computed tomography (CT scan) was performed and showed an evidence of interstitial pulmonary fibrosis. Hiatal hernia and gastric antral vascular ectasia (GAVE) were diagnosed by video capsule endoscopy.

The baseline laboratory tests showed pancytopenia albumin 2.9 g/dL, total bilirubin 1.4 mg/dL, AST 128 U/L and ALT 54 U/L. The total protein was 63.2 g/L.

**Figure 1:**
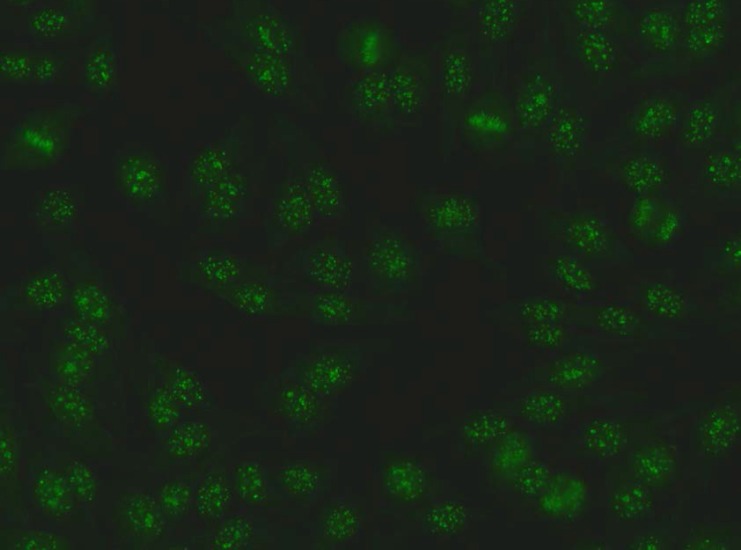
Patient I Hep-2 IIF pattern: Anti-centromere B staining pattern: Rather uniform discrete speckles located throughout the entire nucleus. Telophase and metaphase cells always show these speckles in the condensed chromosomal material

The immunological report revealed positive anti-nuclear antibodies (ANA) 1:1280 with centromeric staining pattern (Figure 1). Anti-mitochondrial (AMA) and liver-kidney microsome type 1 (LKM-1) antibodies were absent, but smooth muscle antibodies were positive (1:320). Second level investigations (Line-ImmunoAssay, LIA Euroimmun) revealed antibodies against CENP-B and SSA/Ro52 KDa. 

Raynaud’s phenomenon was treated with calcium channel blockers and it was currently well controlled. AIH subsided after administration of prednisone, at 40 mg per day for two weeks. After having received 5-10 mg/day of prednisolone as an outpatient and hydroxychloroquine 400mg daily, the patient’s condition remained stable.

**Figure 2:**
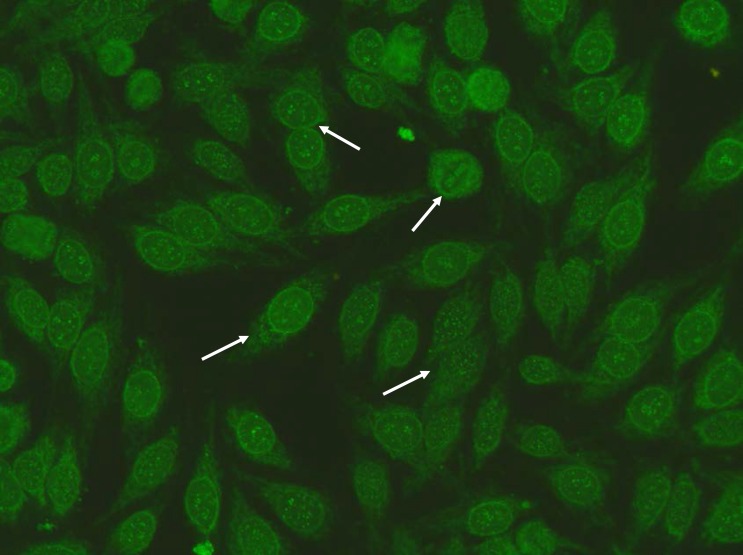
Patient II IIF Hep-2 staining pattern: Anti-centromere A staining pattern: Rather uniform discrete speckles located throughout the entire nucleus. Telophase and metaphase cells always show these speckles in the condensed chromosomal material. Punctate nuclear membranous pattern: focusing through the nucleus can be seen on the surface of the entire nucleus. A similar pattern is seen in telophase and in metaphase the fluorescence is diffusely localized throughout the cytoplasm

**Patient-II**

A 55-year-old white woman was referred to the rheumatology clinic for Raynaud’s phenomenon. She had been diagnosed with AIH, one year before her initial evaluation. During diagnosis, liver function was evaluated as grade A based on the Child-Pugh classification, and International Autoimmune Hepatitis Group score was 16. Viral serologies (hepatitis B surface antigen, anti-hepatitis B surface antigen, immunoglobulin G anti-hepatitis B core antigen, anti-hepatitis C virus, cytomegalovirus, Epstein-Barr virus, herpes simplex virus, and human immunodeficiency virus) were all negative. 

At presentation, she complained of pain in her knees, shoulders, and the metacarpophalangeal joints of both hands. She had Raynaud’s phenomenon, which began approximately 6 to 8 months after the diagnosis of AIH. She presented sclerodactyly, and digital edema characteristic of scleroderma. On examination, she was afebrile with a blood pressure of 130/69 mm Hg and heart rate of 80 per minute. Her physical examination was unremarkable, except for crepitus in both knees. Neither shifting dullness nor fluid wave in abdominal were observed. Capillaroscopy revealed mild tortuosity and dilated capillary loops. 

Pulmonary function tests were within the normal limits with a lung diffusion capacity (DLCO) of 89%.

High resolution CT was performed and there was no evidence of interstitial pulmonary fibrosis. Oesophageal dysmotility was presented and abdominal ultrasonography did not reveal liver cirrhosis or splenomegaly and ascites. 

The baseline laboratory tests showed albumin 3.1 g/dL, total bilirubin 0.8 mg/dL, AST 33 U/L, ALT 35 U/L, and GGT 67 U/L. The total protein was 63.2 g/L.

The immunological report revealed positive anti-nuclear antibodies (ANA) 1:1280 with centromeric nuclear staining pattern and punctate membranous nuclear staining. Diffuse cytoplasmic pattern were also presented (Figure 2). In a strong clinical suspicion of SSc, the identification of specific autoantibodies was necessary. Second-line investigation (Line-Immuno Assay, LIA Euroimmun) revealed specific SSc-related autoantigens, CENP-A and RNA Polymerase III 11 and 155 KDa subunits. The AMA and LKM-1 antibodies were absent, but smooth muscle antibodies were positive (1:320). She currently carries a diagnosis of limited cutaneous systemic sclerosis.

***Literature review***

AIH is an inflammatory liver disease affecting mainly females histologically characterised by interface hepatitis, with abundant lymphocyte and plasma cell infiltrates that cross the limiting plate and invade the liver parenchyma (16), biochemically by elevated transaminase levels and serologically by the presence of autoantibodies. Three types of AIH have been characterized. Type I AIH is the classic syndrome occurring in young women and is associated with marked hypergammaglobulinemia and positive ANA. 

Type II AIH is often seen in children is more common in Mediterranean populations. Type II AIH is associated with liver– kidney microsomal antibodies (anti-LKM), but not with ANA (7). 

Type III AIH occurs more often in women and is associated with positive ANA, SMA, and antibodies to soluble liver antigen/liver pancreas (anti-SLA/LP). Patients with type III AIH are indistinguishable from the patients with classic type I AIH by age, sex distribution, frequency, and nature of other autoantibodies. According to the literature, in Caucasoid population susceptibility resides within the DRB1 gene, and particularly DRB1*0301 is the principal susceptibility allele (7,14). 

While AIH shares clinical and immunological similarities with connective tissue diseases, related reports of AIH to CTD have been scarce. 

Autoimmune hepatitis has been observed in relation to systemic lupus erythematosus (SLE) - accounting for 13% of cases with liver abnormalities, to Sjögren’s syndrome and mixed connective tissue disease, but very rarely to SSc (1,5,14). SSc is a chronic fibrotic immuno-mediated systemic disease that targets the skin, lungs, gastrointestinal tract, kidneys and musculoskeletal system. 

This disorder is clinically characterized by the coexistence of tissue fibrosis, small blood vessel vasculopathy and an autoimmune response associated with specific autoantibodies (17). 

In 1% of a large cohort of patients with SSc, a mild degree of liver involvement was observed while liver fibrosis was found in 9% of patients at autopsy, slightly more prevalent compared with non-SSc controls (18).

The HAI- diagnosis are less frequent in SSc-patients. Abu-Shakra et al. (3) noted that only 4 of 262 SSc patients (1.5%) had a chronic liver disease. In a post-mortem series of 57 SSc patients, D’Angelo et al. (18) found histological liver damage in 8.8% of cases. 

Reviewing the literature, we found eleven case reports of AIH associated to SSc, out of which three cases had AIH-PBC overlap syndrome (5-10, 12,14,15). Limited SSc (9/11) was the most frequent type of SSc; one patient had diffuse SSc and one patient had scleroderma-polymyositis overlap syndrome. With the only exception (15), all patients developed AIH after diagnosis of SSc (5-10, 12,14,15).

Three cases of AIH have been reported in patients with limited cutaneous scleroderma (15). According to two case reports by Marie and co-workers (9), two patients were diagnosed with limited SSc, one to nine years before developing AIH. One patient in Japan had Raynaud’s and developed features of the limited clinical condition, with gangrene and subsequently AIH (6). 

In all three cases, AIH developed after diagnosis of limited cutaneous scleroderma (Table II) (6,9). 

Anticentromere antibodies (ACA) were present in all cases, with one exception (19). 

It is very important to underline that ACA has been detected in 17% of patients with autoimmune hepatitis (7). Anti-mitochondrial antibodies (AMA) were positive in all patients with AIH-PBC overlap syndrome and in one patient without histological features of PBC (12). 

Therapeutic strategies for the treatment of autoimmune liver disease are essentially based on corticosteroids and immunosuppressant drugs such as methotrexate and azathioprine. The exception is provided by PBC, for which ursodeoxycholic acid (UDCA) is the only established treatment (19). With regard to AIH therapy, prednisone and azathioprine were the treatment of choice, with good response. Despite the potential risk of renal crisis in scleroderma during steroid therapy, no cases were reported. Recently, Efe, et al. reported an AIH patient with SSc treated with prednisolone 50 mg/day, azathioprin 50 mg/day, and low-dose angiotensin converting enzyme inhibitor (5). 

## Discussion

The HAI- diagnosis are less frequent in SSc-patients. In fact the chart review of 35 cases with biopsy-proven AIH over the past 5 years at our institution revealed that only two patients developed SSc.

Case report 1 is the first literature that reports an association between AIH and diffuse-SSc sine scleroderma with a contemporary presence of centromeric B protein (CENP-B) and SSa/Ro52 KDa.

There was no evidence for other causes of liver disorders such as alcohol abuse or drugs that could idiosyncratically cause hepatitis, including those that can mimic autoimmune hepatitis.

ACA have been repeatedly demonstrated to be useful biomarkers in the diagnosis of SSc in that they occur in 20 to 40% of these patients and are most commonly associated with the limited cutaneous subset (10,16, 20-22). Although ACA are relatively specific for SSc, they have also been reported in SLE PBC, rheumatoid arthritis (RA) and Sjogren Syndrome (SjS) (22). ACA and other specific SSc-antibodies have historically been considered to be mutually exclusive (21). 

To the best of our knowledge, our second case report describes the first report of a patient with CREST-syndrome/AIH with a contemporary presence of CENP-A/RNA poliemrase III antibodies.

Autoantibodies RNAP III (anti-RNAP III) are highly specific for SSc (22-27). Their prevalence in SSc is approximately 20% and the frequency ranges between 10% to 25% in North American (17, 18), 4% to 31.5% in European (10,19), 5% to 11% in Japanese (21) and 15.3% in Australian (22) patients. Although early studies suggested that anti-RNAP III might be correlated with a speckled nucleolar pattern of IIF staining, a recent study found that antibodies to RNAP-III were not consistently associated with a unique IIF pattern on HEp-2 cell substrates (28). 

This might be explained when it is appreciated that anti-RNAP can overlap with a variety of other autoantibodies. Absolute levels of these biomarkers at baseline or during the disease course do not predict organ complications or disease outcome. Although, there was some correlation with the severity of skin involvement (28). 

Anti-Ro/SSa52 specificity is frequent in autoimmune liver diseases. Granito, et al. (29) found that it was the most frequent anti-ENA reactivity in primary biliary cirrhosis (PBC, 28%), and that anti-Ro/SSA52 were in a more advanced histological stage and had higher serum levels of bilirubin and IgM at the time of diagnosis. Liaskos, et al. (30) found that the serum from patients with AIH I, and soluble liver antigen autoantibodies (anti-SLA) simultaneously reacts to Ro52 autoantigen. Ninety-eight percent of the anti-SLA-positive serum samples reacted with SSA/Ro52 and were associated with biochemically and histologically more severe disease than those taken from tRNP(Ser)Sec-negative AIH I patients with or without anti-SSA/Ro52 antibodies (31). 

The pathological mechanisms of autoimmune hepatitis in SSc patients remain unclear, which raises the question of whether the condition resulted from a causal association or by chance (32). However, autoimmune hepatitis may be due in part to activation of the cellular and humoral immune responses, as nearly all SSc patients are antinuclear– antibody positive. In fact circulating CD4+T cells have elevated levels of chemokine receptors and express alpha 1 integrin (an adhesion molecule), accounting for their enhanced ability to bind to endothelium and to fibroblasts (32). Activated macrophages and T-cells can induce TGF-beta, a powerful modulator of immune regulation and matrix accumulation (32). 

Based on our observation and literature review, we suggest that SSc may be considered to be one of the manifestations associated with AIH. Patients with AIH may be at increased risk for developing SSc and should be followed closely. 

Should individuals with autoimmune diseases be considered the tip of the iceberg, or as the first manifestation of a systemic disease? The re-assessment of diagnostic protocols and precise observation of the clinical picture will certainly help to clarify the nature of the pathogenesis of the individual diseases and the aetiology of deregulation of the immune network.
